# Alterations in the Expression of Tight and Adherens Junction Proteins in the Hen Oviduct Following Tamoxifen Treatment

**DOI:** 10.3390/ijms27219738

**Published:** 2026-10-30

**Authors:** Karolina Frydrych, Dominika Wolak, Anna Hrabia

**Affiliations:** Department of Animal Physiology and Endocrinology, University of Agriculture in Krakow, 31-120 Krakow, Poland; karolina.frydrych@student.urk.edu.pl (K.F.); dominika.wolak@urk.edu.pl (D.W.)

**Keywords:** tight junction, adherens junction, tamoxifen, oviduct, hen

## Abstract

Intercellular junctions are essential for maintaining epithelial integrity, barrier function, and tissue remodeling in the female reproductive system. However, their role in the avian oviduct is poorly understood. The aim of this study was to examine the effect of tamoxifen (TMX; an estrogen receptor blocker in birds) treatment on the histoarchitecture and the abundance of transcripts and proteins, as well as the immunolocalization of key tight junction proteins (occludin, claudin 1, and ZO1) and adherens junction proteins (E-cadherin and β-catenin) in the oviduct of hens. Tissue samples were collected from all oviductal segments (i.e., the infundibulum, magnum, isthmus, shell gland, and vagina) on day 8 of the experiment, from control (laying; n = 6) hens and hens treated (6 mg/kg body weight) daily with TMX (non-laying; n = 6). Following TMX treatment for 7 consecutive days, a pause in laying and partial oviductal regression was accompanied by segment-dependent alterations in mRNA transcript abundances (examined by qRT-PCR) and/or protein abundances (determined by Western blot) of the examined junctional molecules. Furthermore, TMX treatment altered the patterns of immunofluorescent staining within the oviductal wall, concomitant with the disorganization of the luminal epithelium and tubular glands. Our results suggest that the expression and localization of tight and adherens junction proteins in the hen oviduct depend strongly on estrogen signaling. Moreover, this study provides novel insights into the regulation of these proteins, indicating their potential involvement in the molecular mechanisms that orchestrate epithelial remodeling and functioning in the hen oviduct, which may affect poultry production.

## 1. Introduction

In adult laying hens, the oviduct is a specialized organ responsible for egg formation, involving the deposition of proteins around the yolk and the formation of a calcified eggshell. It is composed of five morphologically and functionally different regions: the infundibulum, magnum, isthmus, shell gland (uterus), and vagina. Egg formation is a highly coordinated process lasting approximately 24–25 h [1]. Following ovulation, the oocyte is captured by the infundibulum, where fertilization occurs and the first layer of albumen is deposited. The magnum produces the majority of egg albumen, whereas the isthmus is responsible for the formation of eggshell membranes [2]. The shell gland mediates eggshell formation through calcium carbonate deposition [3]. The vagina does not participate directly in egg formation but rather facilitates the expulsion of the egg from the oviduct (oviposition) and serves as a sperm storage site within the sperm storage tubules located at the utero-vaginal junction [4].

The development and function of the oviduct are closely associated with the reproductive cycle and are regulated by the interaction of various hormones, growth factors, and neurotransmitters. Ovarian steroids, particularly estrogen, play a crucial role in this process [1,5]. Estradiol is a key regulator of development, function, and remodeling of the oviduct, including its regression and rejuvenation after molting. Estrogen exerts its effects through the classical nuclear estrogen receptors, ERα and ERβ [6,7], as well as the membrane-associated G-protein-coupled estrogen receptor (GPER) [8], thereby regulating essential processes for epithelial differentiation, secretory activity, and tissue homeostasis.

Tamoxifen (TMX) is a non-steroidal, selective estrogen receptor modulator (SERM) which binds competitively to estrogen receptors, blocking estradiol-dependent transcriptional activity in target tissues [9], including the avian reproductive system. In birds, TMX binds to ERs with high affinity [10], and only its an antagonistic effect [10,11] was shown. Therefore, this compound has been frequently used in studies of the physiological role of estrogens [12,13,14,15]. The continuous treatment of TMX to laying hens leads to a lowering of the concentration of plasma progesterone in hens without changes in concentrations of testosterone and estradiol [12,16]. Furthermore, TMX treatment may abolish the action of estradiol at the hypothalamic–pituitary axis by decreasing progesterone receptor abundance, leading to inhibition of the preovulatory surge of luteinizing hormone in response to progesterone [17,18]. The blocking of ERs by TMX also causes the induction of cell apoptosis, regression of the reproductive system, and cessation of egg laying, making it a well-established experimental model for studying estrogen-dependent regulation of reproductive tissues [12,13,15,16,17,19].

The epithelial layer of the hen oviduct is characterized by intense secretory activity and is a key structural and functional component in egg formation [1,3]. The integrity of this epithelium depends on the regulation of intercellular communication and adhesion, which is mediated by specialized junctional proteins. In particular, tight junctions and adherens junctions play a fundamental role in preserving epithelial polarity, barrier function, and mechanical cohesion [20,21,22,23,24].

Intercellular communications in epithelial cells are mediated by three main types of junctions: tight junctions, adherens junctions, and gap junctions. Tight junctions are multiprotein complexes located at the apical region of epithelial cells, forming a seal that regulates paracellular permeability. They are composed of transmembrane proteins, such as primarily occludin (encoded by the *OCLN* gene) and members of the claudin family (encoded by the *CLDN* gene), which are functionally associated with intracellular proteins of the zonula occludens family (ZOs, also known as tight junction protein [TJP]), which ensure the organization of junctions and the regulation of barriers [25,26,27,28].

Adherens junctions are primarily responsible for maintaining epithelial cohesion, supporting the cytoskeleton, and facilitating intracellular signaling. Their core molecular components include cadherins and catenins, which mediate cell–cell adhesion and connect junctional complexes to the actin cytoskeleton [24,29].

Numerous studies have demonstrated the importance of intercellular junctions and their constituent proteins in the regulation of reproductive functions in mammals [30,31,32,33,34,35,36]. Moreover, it has been confirmed in mammalian models that estrogen is a key regulator of junctional integrity [37,38,39]. However, studies on the role of cell–cell junctions, as well as the expression, localization, and hormonal regulation of these junction proteins in the oviduct of hens, are scarce. So far, it was demonstrated that estradiol increases the expression of claudin-1, -3, and -5 in the chicken oviduct, whereas plasma estradiol decline during molting decreases their levels and impairs mucosal barrier function [40]. More recently, the expression of several connexins (Cxs), gap junction proteins, has been shown to be segment-specific in the hen oviduct [19]. In addition, alterations in the expression of the Cx43 gene and protein in the hen oviduct following TMX treatment have been demonstrated [19], indicating the estrogen-dependent regulation of intercellular communication in tissues of the avian oviduct.

Given the fundamental roles of tight and adherens junctions in maintaining epithelial integrity and regulating paracellular permeability, as well as their potential implication in supporting processes of egg formation, and, on the other hand, the possible role of estradiol in cell–cell junction protein regulation, we hypothesized that gene and protein expression of tight and adherens junction constituents in the hen oviduct is affected by TMX (estrogen receptor blocker in birds) treatment. Accordingly, the aim of the study was to examine: (1) the tissue structure and mucosal barrier integrity by determining the histoarchitecture and (2) the abundances of transcripts and proteins and the immunolocalization of key tight (occludin, claudin 1, ZO1) and adherens (E-cadherin, β-catenin) junction proteins in the oviduct of hens subjected to a pause in laying by TMX treatment.

## 2. Result

### 2.1. Changes in the Histoarchitecture of the Hen Oviductal Wall After TMX Treatment

**Figure 1:**
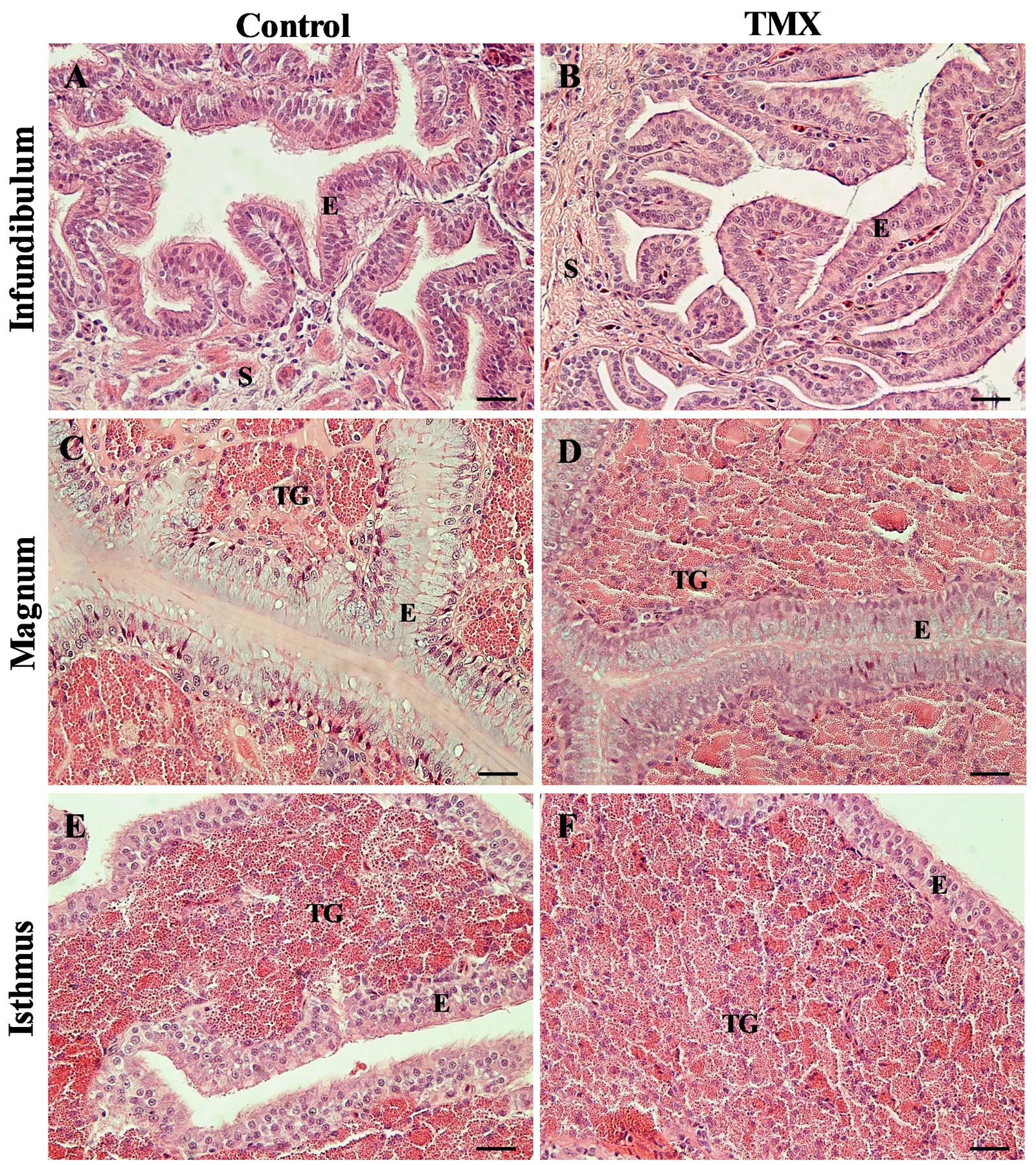

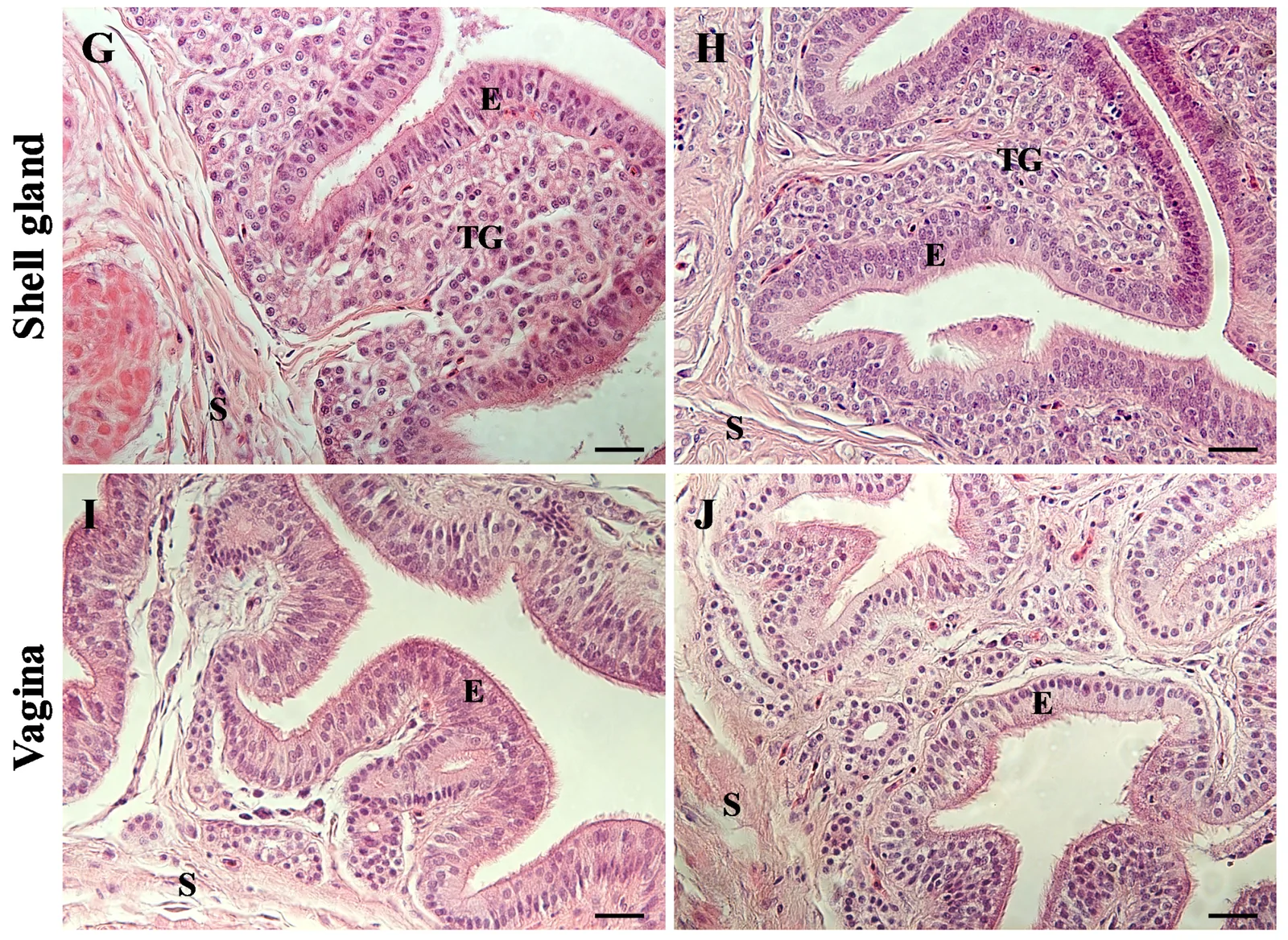
Representative histological images of the hen oviductal wall after tamoxifen (TMX) treatment; visualized by hematoxylin and eosin (**E**,**H**) staining. Micrographs represent the infundibulum (**A**,**B**), magnum (**C**,**D**), isthmus (**E**,**F**), shell gland (**G**,**H**), and vagina (**I**,**J**). In the control group, the normal architecture of the luminal epithelium (E), tubular glands (TG), and stroma (S; muscles + connective tissue) is clearly visible. In TMX-treated (non-laying) hens, the luminal epithelium appears thinner and is accompanied by a reduction, involution, and degeneration of the tubular glands in the magnum, isthmus, and shell gland. The stroma remains preserved in both experimental groups. Scale bars = 20 μm.

**Figure 2:**
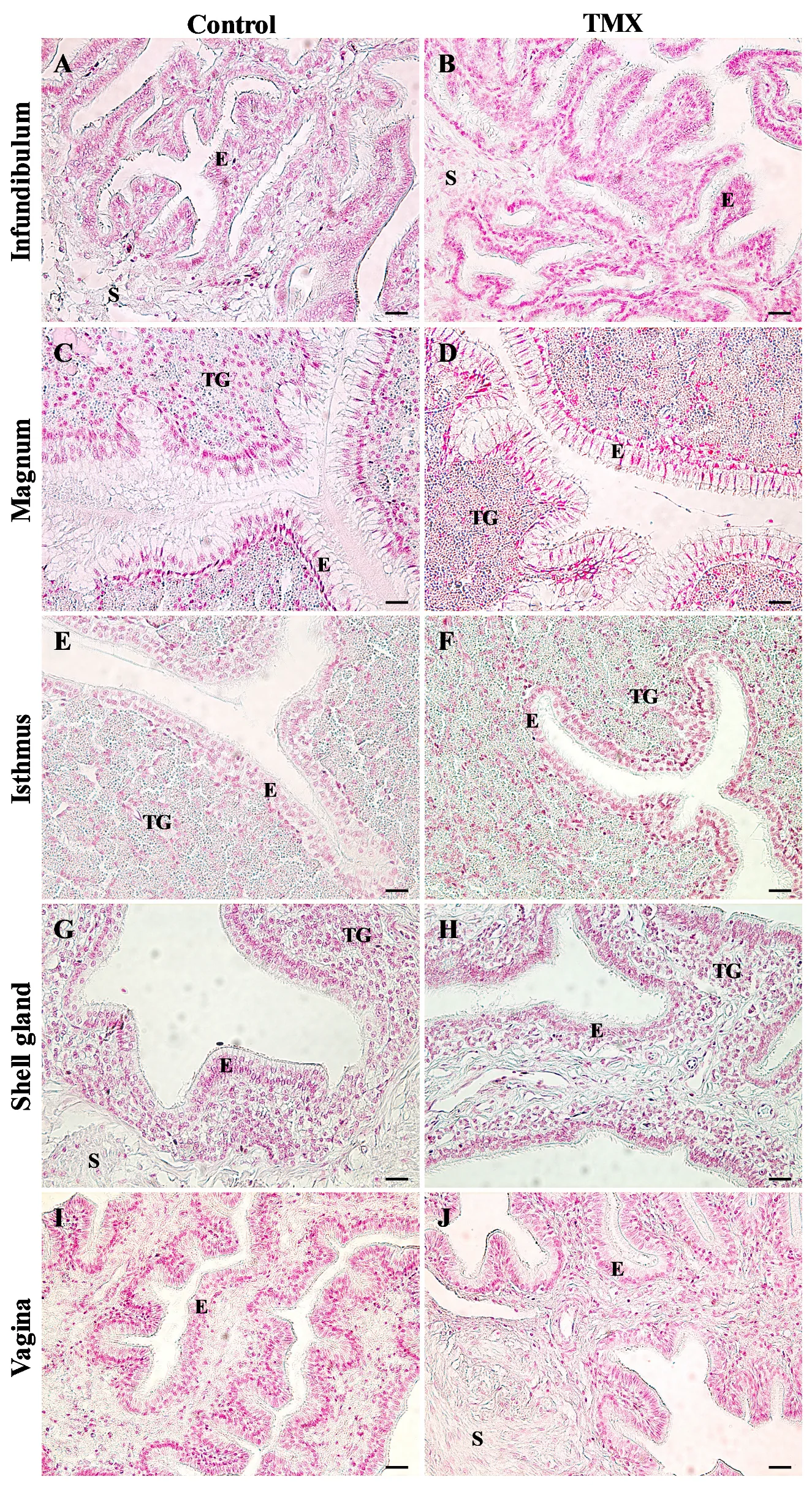
Histological changes in the oviduct wall of hens after tamoxifen (TMX) treatment; visualized by Feulgen staining. Micrographs demonstrate tissue sections of different segments of the oviduct: the infundibulum (**A**,**B**), magnum (**C**,**D**), isthmus (**E**,**F**), shell gland (**G**,**H**), and vagina (**I**,**J**). Compared to the control hens, a noticeable thinning of the luminal epithelium (E) and regression of the tubular glands (TG) are evident in the oviduct of hens subjected to TMX treatment. The pink-stained DNA highlights differences in the cellular structure. In TMX-treated birds, the presence of fragmented or smaller cell nuclei is observed, which may indicate involution processes, particularly in the magnum and isthmus. The stroma (S; muscles + connective tissue) remains unchanged. Bars = 50 μm.

Changes in the structure of the oviductal wall following TMX treatment, assessed by H&E and Feulgen staining, are presented in Figure 1 and Figure 2, respectively. Histological analysis based on H&E staining revealed that the luminal epithelium, tubular glands, and stroma were well-preserved in control hens across all oviductal segments.

**Figure 3:**
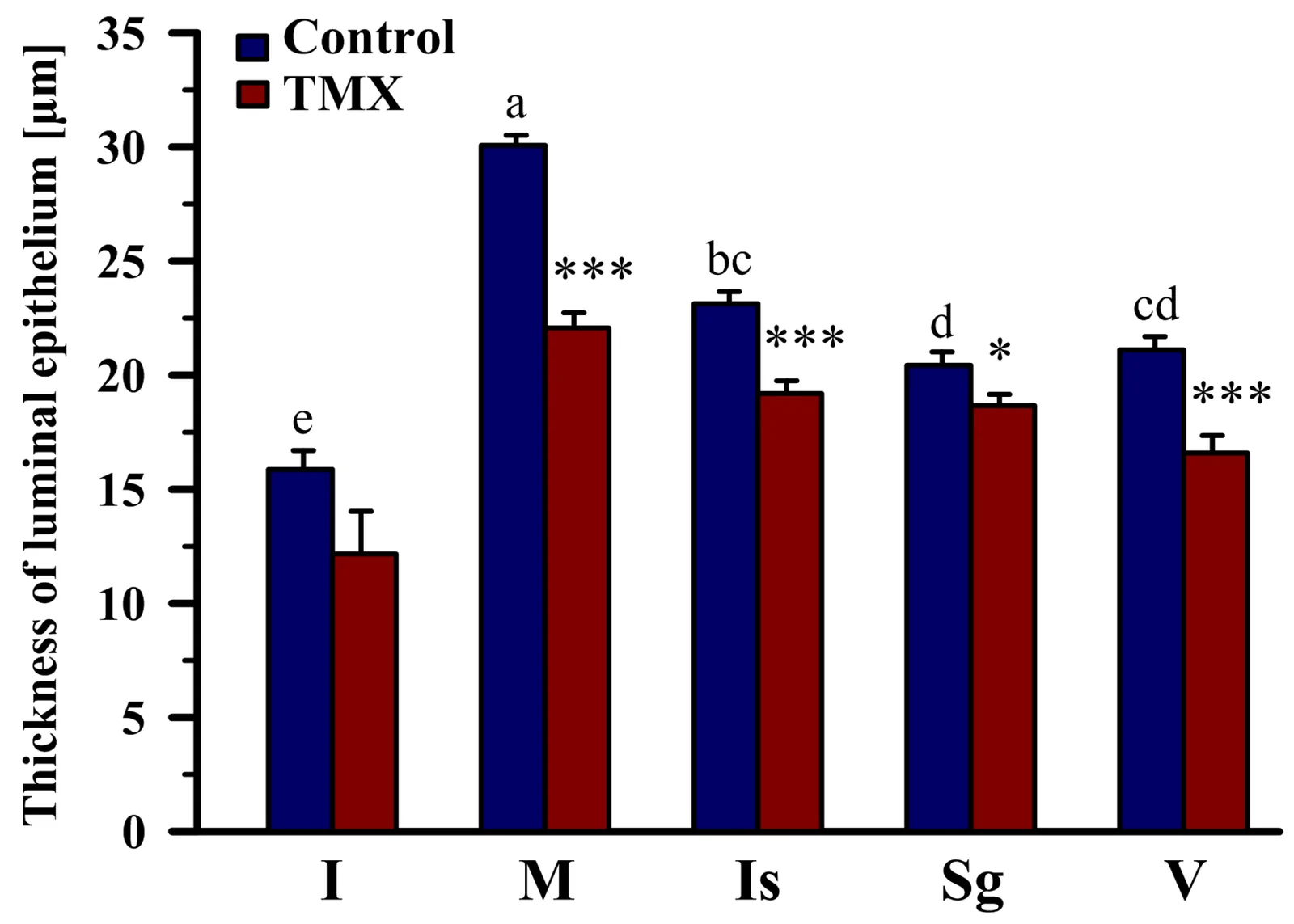
Thickness of the luminal epithelium [μm] in oviductal segments of control and tamoxifen (TMX)-treated hens. Each bar represents the mean ± SEM from six different hens/biological replicates. Values marked with different letters differ significantly (*p* < 0.001; one-way ANOVA followed by the Tukey test) in the control group. * *p* < 0.05, *** *p* < 0.001—vs. control (Student’s *t*-test). Abbreviations: I, infundibulum; M, magnum; Is, isthmus; Sg, shell gland; V, vagina.

In TMX-treated hens, a significant reduction in the thickness of the luminal epithelium was observed in the magnum, isthmus, shell gland, and vagina by 27% (*p* < 0.001), 17% (*p* < 0.001), 9% (*p* = 0.046), and 21% (*p* < 0.001), respectively, compared to control hens. In the infundibulum, there was only a tendency (*p* = 0.099) for the thickness to decrease (Figure 3).

Disorganization and partial involution of the tubular glands, present in the magnum, isthmus, and shell gland, were also observed. The stroma appeared morphologically unchanged after TMX treatment (Figure 1). Feulgen staining confirmed partial regression of the oviductal tissues of TMX-treated hens, including a reduction in the height of the luminal epithelium and the involution of the tubular glands. Furthermore, numerous condensed and fragmented nuclei were observed in both the luminal epithelium and the tubular glands throughout the oviduct (Figure 2).

### 2.2. Expression of mRNA for ZO1, CDH1, and CTNNB1 in the Hen Oviduct After TMX Treatment

**Figure 4:**
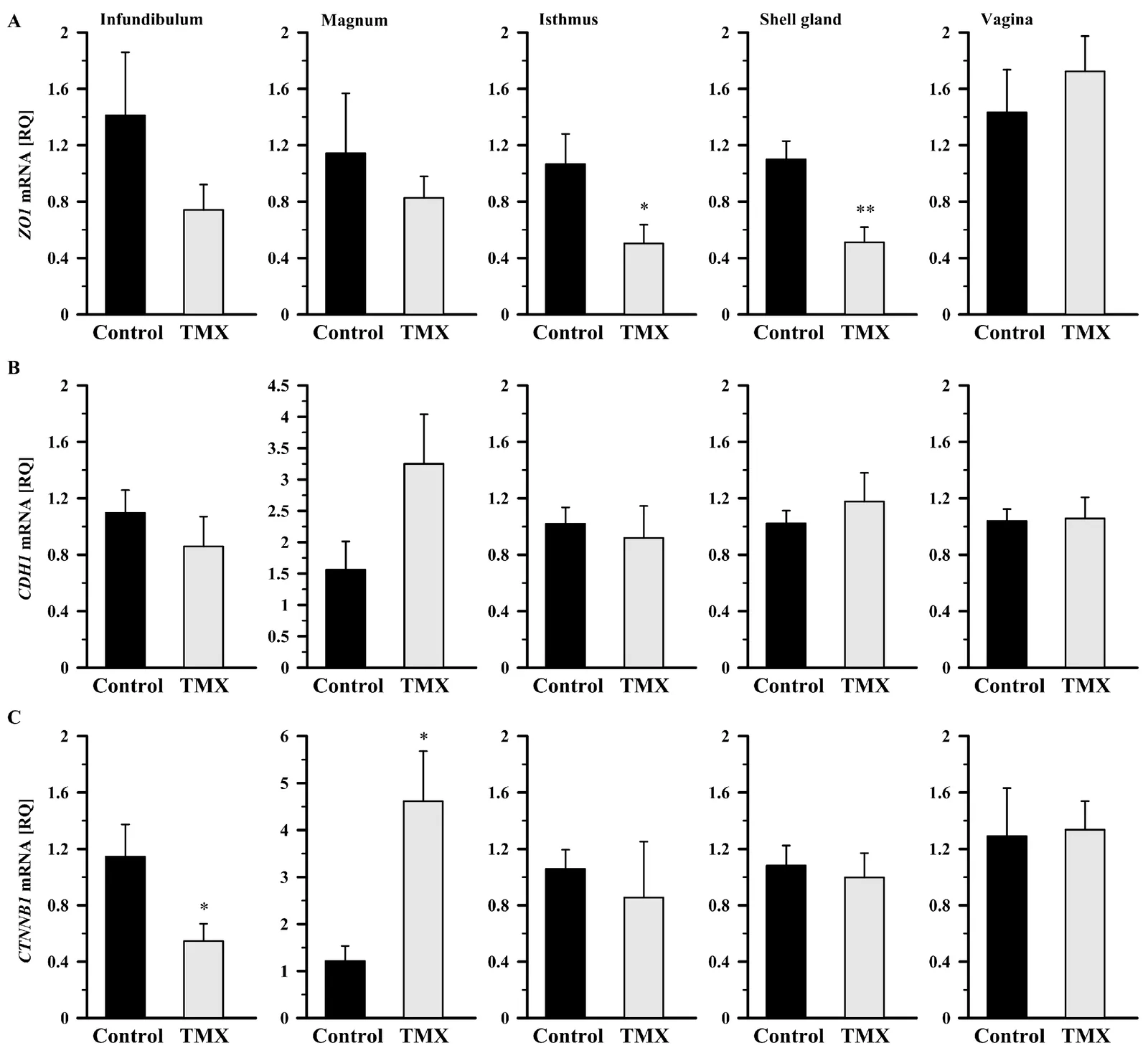
Relative gene expression of *ZO1* (**A**), *CDH1* (**B**), and *CTNNB1* (**C**) mRNA in oviductal segments of control and tamoxifen (TMX)-treated hens. Each value represents the mean relative quantity (RQ) ± SEM from five different hens/biological replicates, normalized to *18S rRNA* and standardized to the expression in tissues of control hens. Asterisks denote significant differences between control and TMX-treated hens for each segment (* *p* < 0.05, ** *p* < 0.01; Mann–Whitney U test). Abbreviations: *ZO1*, zonula occludens 1; *CDH1*, cadherin 1; *CTNNB1*, catenin beta-1 (also known as β-catenin).

As shown in Figure 4, TMX treatment caused a 52.9% decrease in *ZO1* mRNA transcript abundance in the isthmus (*p* = 0.041) and a 53.5% decrease in the shell gland (*p* = 0.003), compared to control hens. No significant differences in *CDH1* gene expression were found in oviductal segments following TMX treatment (*p* > 0.05), but TMX treatment caused a 52.3% decrease in *CTNNB1* mRNA levels (*p* = 0.027) in the infundibulum and a 279.3% increase (*p* = 0.020) in the magnum, compared to control hens.

### 2.3. The Abundance of Tight Junction Proteins (Occludin, Claudin 1, and ZO1) in the Hen Oviduct After TMX Treatment

**Figure 5:**
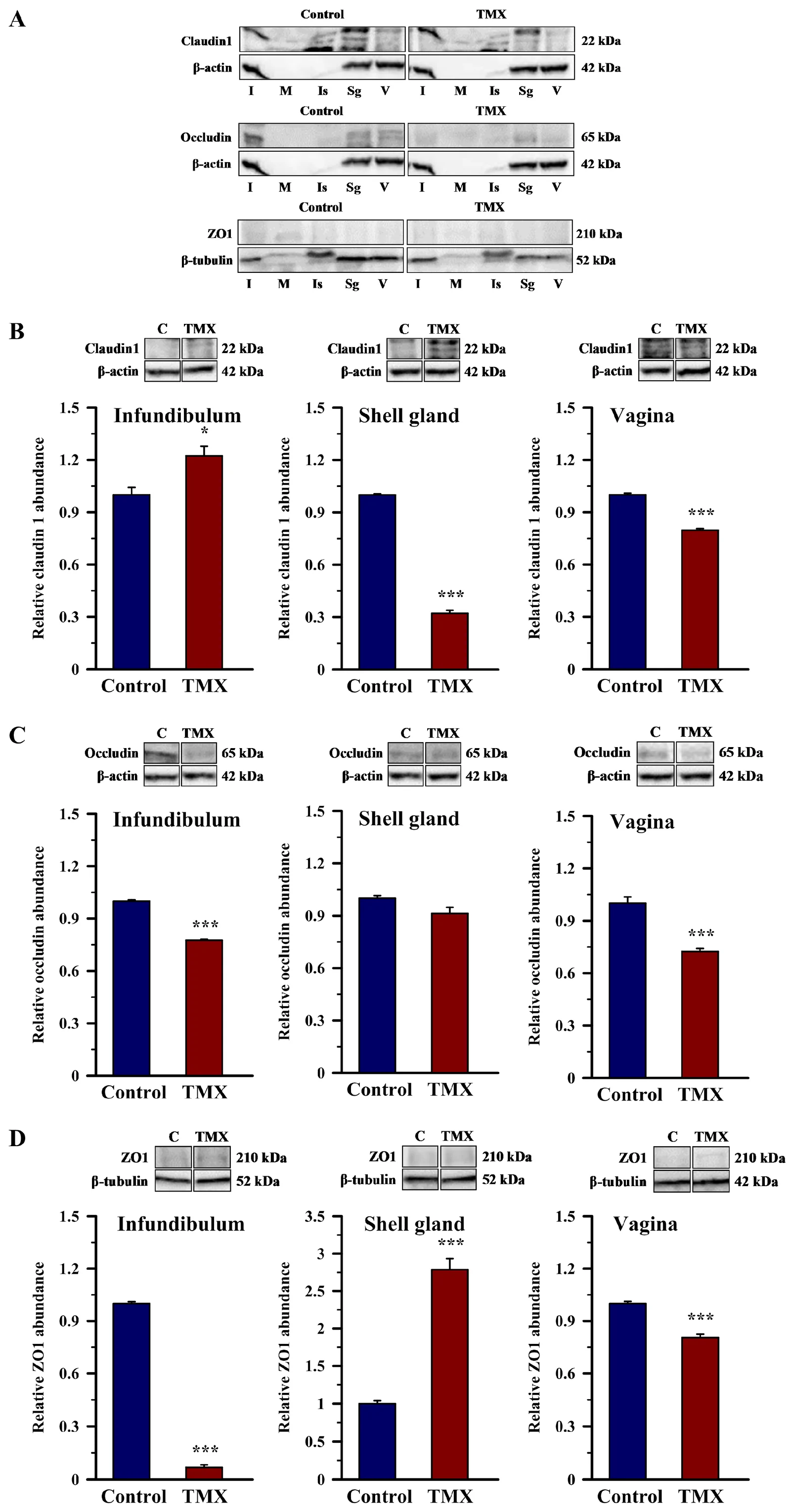
Western blot analysis of tight junction protein (claudin 1, occludin, and ZO1) abundance in oviductal parts of control and tamoxifen (TMX)-treated hens evaluated by Western blot. (**A**) Representative blots of claudin 1, occludin, and ZO1 in all oviductal segments of control and TMX-treated hens. β-actin or β-tubulin were used as loading controls. (**B**–**D**) The blots and relative abundances of claudin 1 (**B**), occludin (**C**), and ZO1 (**D**) proteins in the infundibulum, shell gland, and vagina of the control and TMX-treated hens. Upper panels are representative of four replicates (tissues from six different hens/biological replicates per group) performed for each protein. Graphs present the relative abundances normalized to β-actin or β-tubulin. Data are expressed as fold difference ± SEM (n = four replicates) and compared to the abundance in the control group, considered to be 1. * *p* < 0.05, *** *p* < 0.001—compared to the control tissue (Mann–Whitney U test). Abbreviations: ZO1: zonula occludens 1; I, infundibulum; M, magnum; Is, isthmus; Sg, shell gland; V, vagina; C, control; TMX, tamoxifen.

Due to the limited availability of commercial antibodies against chicken cell–cell junction proteins, Western blot analyses were performed only for selected tight junction proteins in different segments of the hen oviduct (Figure 5). Immunoreactive bands corresponding to claudin 1 (~22 kDa), occludin (~65 kDa), and ZO1 (~210 kDa) were detected in all analyzed oviductal segments from both control and TMX-treated hens (Figure 5A). However, due to the low or undetectable abundance of the reference proteins (β-actin and β-tubulin) in some oviductal segments, densitometric analysis could only be performed for the infundibulum, shell gland, and vagina. The relative abundance of claudin 1, occludin, and ZO1 proteins in the analyzed oviductal segments is shown in Figure 5B–D.

Following TMX treatment, claudin 1 protein abundance increased by 22.3% (*p* = 0.019) in the infundibulum, while a significant decrease was observed in the shell gland and vagina by 67.8% (*p* < 0.001) and 20.3% (*p* < 0.01), respectively (Figure 5B). Occludin protein abundance decreased following TMX treatment by 22.4% (*p* < 0.001) in the infundibulum and by 27.6% (*p* < 0.01) in the vagina, compared to control hens (Figure 5C). TMX treatment also affected ZO1 protein abundance: in the infundibulum and vagina, ZO1 abundance decreased by 93.2% (*p* < 0.001) and 19.5% (*p* < 0.001), respectively, while in the shell gland, it increased by 178.8% (*p* < 0.001) relative to the control group (Figure 5D).

### 2.4. The Abundance of Adherens Junction Proteins (E-Cadherin and β-Catenin) in the Hen Oviduct After TMX Treatment

**Figure 6:**
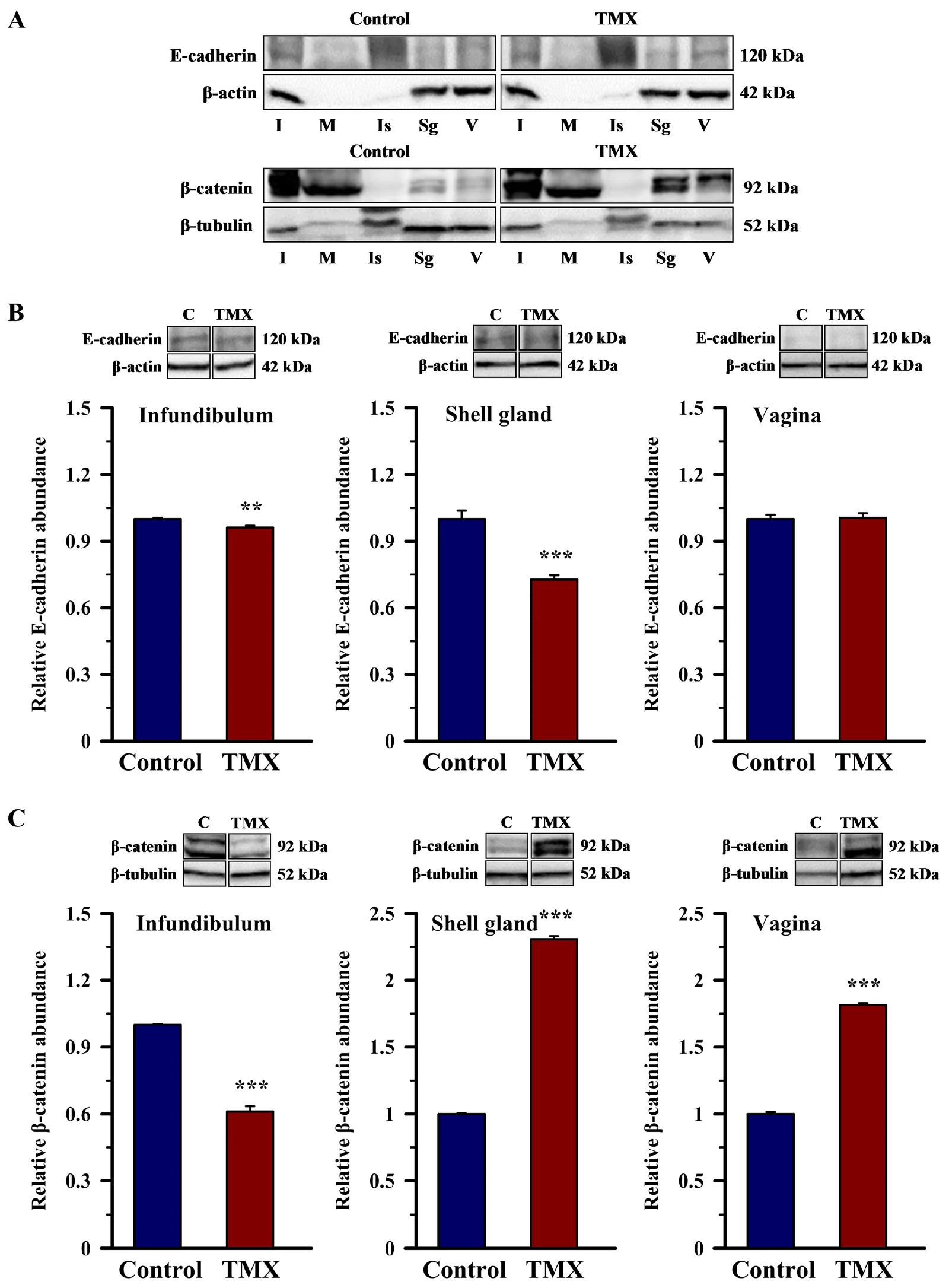
Western blot analysis of adherens junction protein (E-cadherin and β-catenin) abundance in oviductal parts of control and tamoxifen (TMX)-treated hens evaluated by Western blot. (**A**) Representative blots of E-cadherin and β-catenin in all oviductal segments of control and TMX-treated hens. β-actin or β-tubulin were used as loading controls. (**B**,**C**) The blots and relative abundances of E-cadherin (**B**) and β-catenin (**C**) proteins in the infundibulum, shell gland, and vagina of control and TMX-treated hens. Upper panels are representative of four independent replicates (tissues from six different hens/biological replicates per group) performed for each protein. Graphs present the relative abundances normalized to β-actin or β-tubulin. Data are expressed as fold difference ± SEM (n = four replicates) and compared to the abundance in the control group, considered to be 1. ** *p* < 0.01, *** *p* < 0.001—compared to the control tissue (Mann–Whitney U test). Abbreviations as in Figure 4.

Due to the limited availability of commercial antibodies against chicken intercellular junction proteins, Western blot analyses were only performed for E-cadherin and β-catenin in different segments of the hen oviduct (Figure 6). Immunoreactive bands corresponding to ~ 120 kDa and ~ 92 kDa, respectively, were detected for E-cadherin and β-catenin in all analyzed samples from both control and TMX-treated hens (Figure 6A). However, due to the low or undetectable abundance of the analyzed proteins, as well as the reference proteins (β-actin and β-tubulin), in the magnum and isthmus, densitometric analysis could only be performed on the infundibulum, shell gland, and vagina. The relative abundance of the analyzed proteins in these segments is presented in Figure 6B,C.

Following TMX treatment, the abundance of adherens junction proteins was altered in the analyzed oviductal segments. E-cadherin protein abundance decreased by 3.8% (*p* = 0.006) in the infundibulum and by 27.3% (*p* < 0.001) in the shell gland compared to the control hens (Figure 6B). The abundance of β-catenin protein, with both detected forms analyzed together, also differed following TMX treatment. In the infundibulum, β-catenin protein abundance decreased by 38.9% (*p* < 0.001), whereas in the shell gland and vagina, it increased by 130.8% (*p* < 0.001) and 81.5% (*p* < 0.001), respectively, relative to the control group (Figure 6C).

### 2.5. Immunofluorescent Localization of Claudin 1, Occludin, and ZO1 Proteins in the Hen Oviduct After TMX Treatment

**Figure 7:**
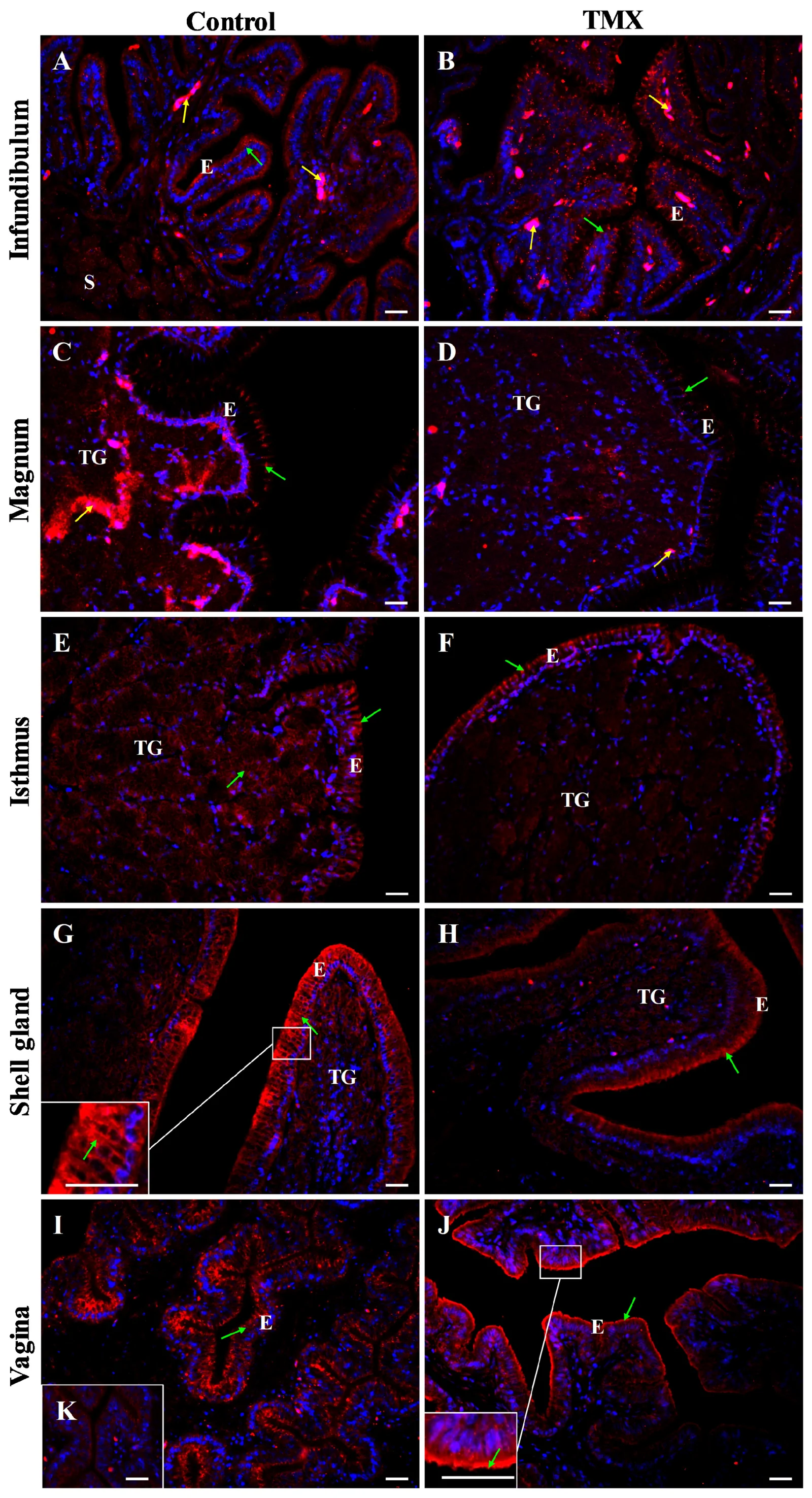
Immunofluorescent localization of claudin 1 protein in oviductal segments of control hens (**A**,**C**,**E**,**H**,**J**) and tamoxifen (TMX)-treated hens (**B**,**D**,**F**,**H**,**J**). A positive reaction (red) is indicated by green arrows, while non-specific staining in erythrocytes is marked by yellow arrows. DAPI staining (blue) shows cell nuclei. The frames in (**G**,**J**) indicate the location of a higher magnification view (insets). (**K**) Negative control (inset in (**I**)). Compared to the controls, the TMX-treated hens showed diminished claudin-1 immunoreactivity, with an irregular distribution and loss of distinct apical localization. Abbreviations: E: luminal epithelium; S: stroma (muscles + connective tissue); TG: tubular glands. Scale bars = 20 μm.

**Figure 8:**
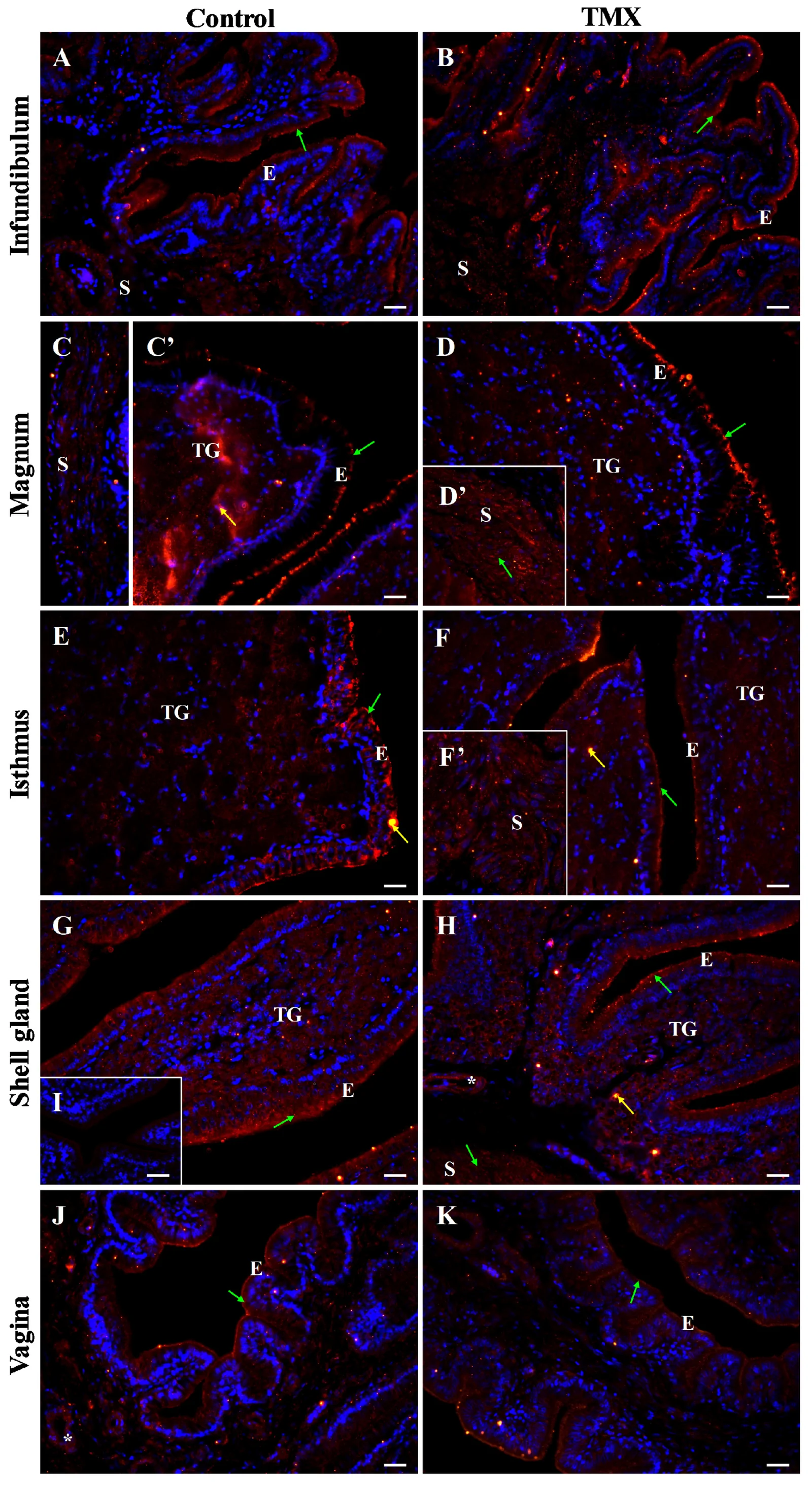
Immunofluorescent localization of occludin protein in oviductal segments of control hens (**A**,**C**,**C′**,**E**,**G**,**J**) and tamoxifen (TMX)-treated hens (**B**,**D**,**D′**,**F**,**F′**,**H**,**K**). Positive immunoreactive signals (red) are indicated by green arrows; non-specific staining in erythrocytes is marked by a yellow arrow. DAPI staining (blue) indicates cell nuclei. Inserts (**D′**,**F′**) show the oviductal stroma (S). (**I**) Inset in (**G**) shows the negative control. Of note, the intensity of the immunopositive reaction for occludin in the oviduct of the TMX-treated hens is reduced and staining is more diffuse and irregular compared to the control hens. Abbreviations: *, blood vessel. Other abbreviations as in Figure 6. Scale bars = 20 μm.

**Figure 9:**
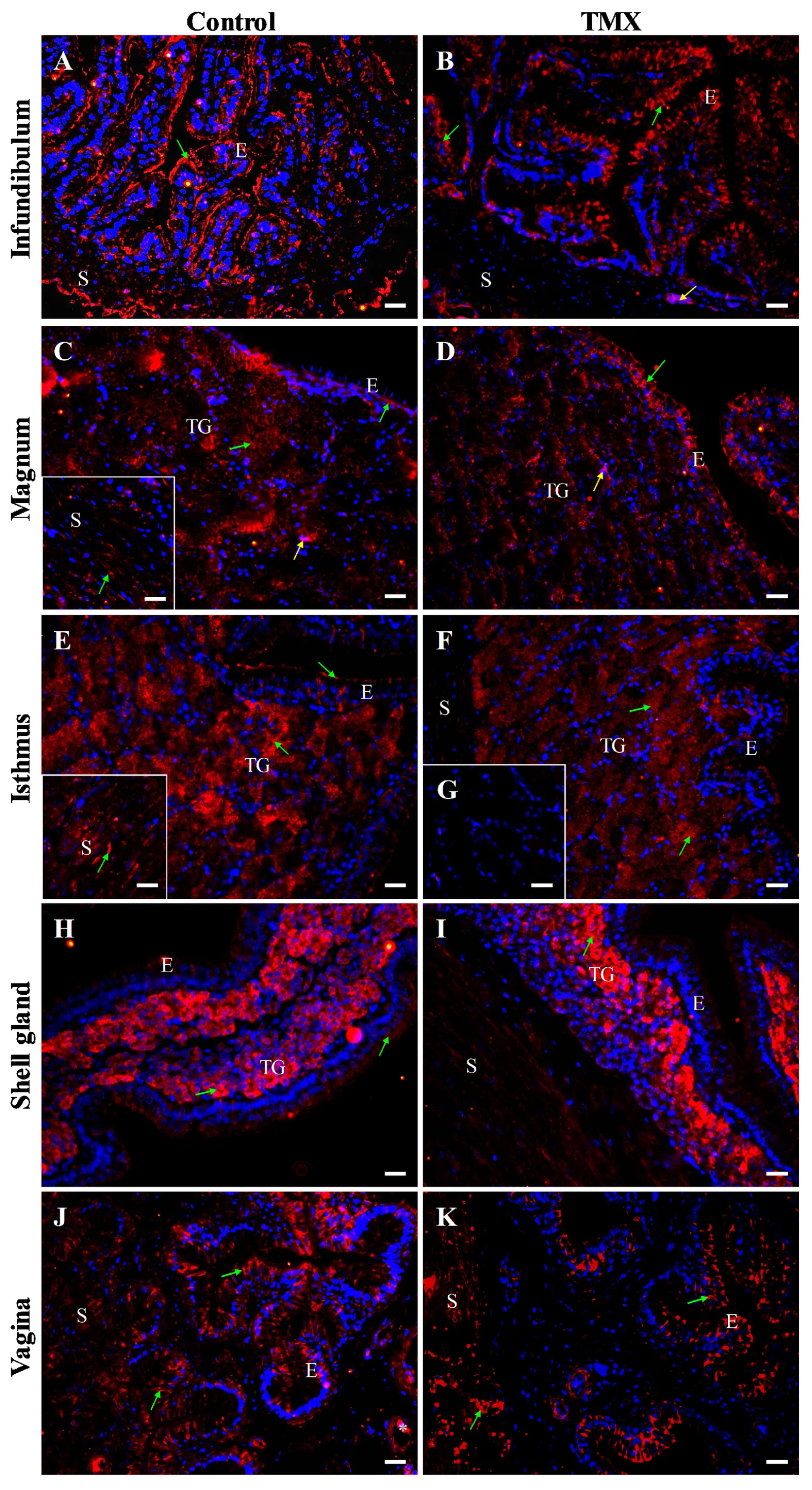
Immunofluorescent localization of ZO1 protein in oviductal segments of control hens (**A**,**C**,**E**,**H**,**J**) and TMX-treated hens (**B**,**D**,**F**,**I**,**K**). Positive immunoreactive signals (red) are indicated by green arrows, non-specific staining in erythrocytes is marked by yellow arrows. DAPI staining (blue) indicates cell nuclei. The insets in C and E show the oviductal stroma of control hens. (**G**) Inset in F shows the negative control. In TMX-treated hens, ZO1 signal intensity was diminished and disorganized. Abbreviations: *, blood vessel. Other abbreviations as in Figure 6. Scale bars = 20 μm.

Consistent with the Western blot results, immunofluorescent analysis confirmed the presence of claudin 1, occludin, and ZO1 proteins in all oviductal segments (Figure 7, Figure 8 and Figure 9). In control hens, claudin 1 immunoreactivity was detected in all oviductal segments (Figure 7), with strong immunopositive signals visible in the membranes of adjacent cells of the luminal epithelium in the shell gland and vagina. By contrast, weak immunoreactivity was detected in the infundibulum, magnum, and isthmus. Weak linear and punctate signals were also present in tubular glands of the magnum, isthmus, and shell gland. Following TMX treatment, claudin 1 immunoreactivity in the luminal epithelium of the infundibulum was stronger compared to the control. In the magnum, isthmus, and shell gland, the intensity of immunopositive signals decreased in the luminal epithelium and/or the tubular glands. Furthermore, claudin 1 immunoreactivity became less distinct and more fragmented, with signals frequently redistributed towards the apical region of luminal epithelial cells.

Occludin immunoreactivity was detected in all oviductal segments (Figure 8). In control hens, moderate or weak immunopositive signals appeared as distinct punctate foci or irregular lines, localized between adjacent epithelial cells, mainly on the apical side. Weak, punctate immunoreactivity was also observed in the tubular glands and smooth muscle layers. Following TMX treatment, the intensity of occludin immunoreactivity was slightly reduced in the luminal epithelium of the isthmus and vagina compared to the control group. The signals appeared more dispersed and irregular and frequently shifted towards the cytoplasm.

ZO1 immunoreactivity was detected in all oviductal segments (Figure 9). In control hens, strong immunopositive signals appearing as distinct punctate or linear structures were observed in the luminal epithelium of the infundibulum and vagina, as well as in tubular glands of the isthmus and shell gland. Moderate ZO1 immunoreactivity was present in the tubular glands of the magnum and in smooth muscles of the oviduct. Following TMX treatment, both the intensity and distribution of ZO1 immunoreactivity were altered. In the magnum and isthmus, signals in the tubular gland were weaker. In contrast, ZO1 immunoreactivity in the tubular glands of the shell gland was slightly more intense following TMX treatment.

Negative control sections incubated without the primary antibody showed no immunopositive signals for occludin, claudin 1, or ZO1 (inserts in Figure 7J, Figure 8K and Figure 9G).

### 2.6. Immunofluorescent Localization of E-Cadherin and β-Catenin Proteins in the Hen Oviduct After TMX Treatment

**Figure 10:**
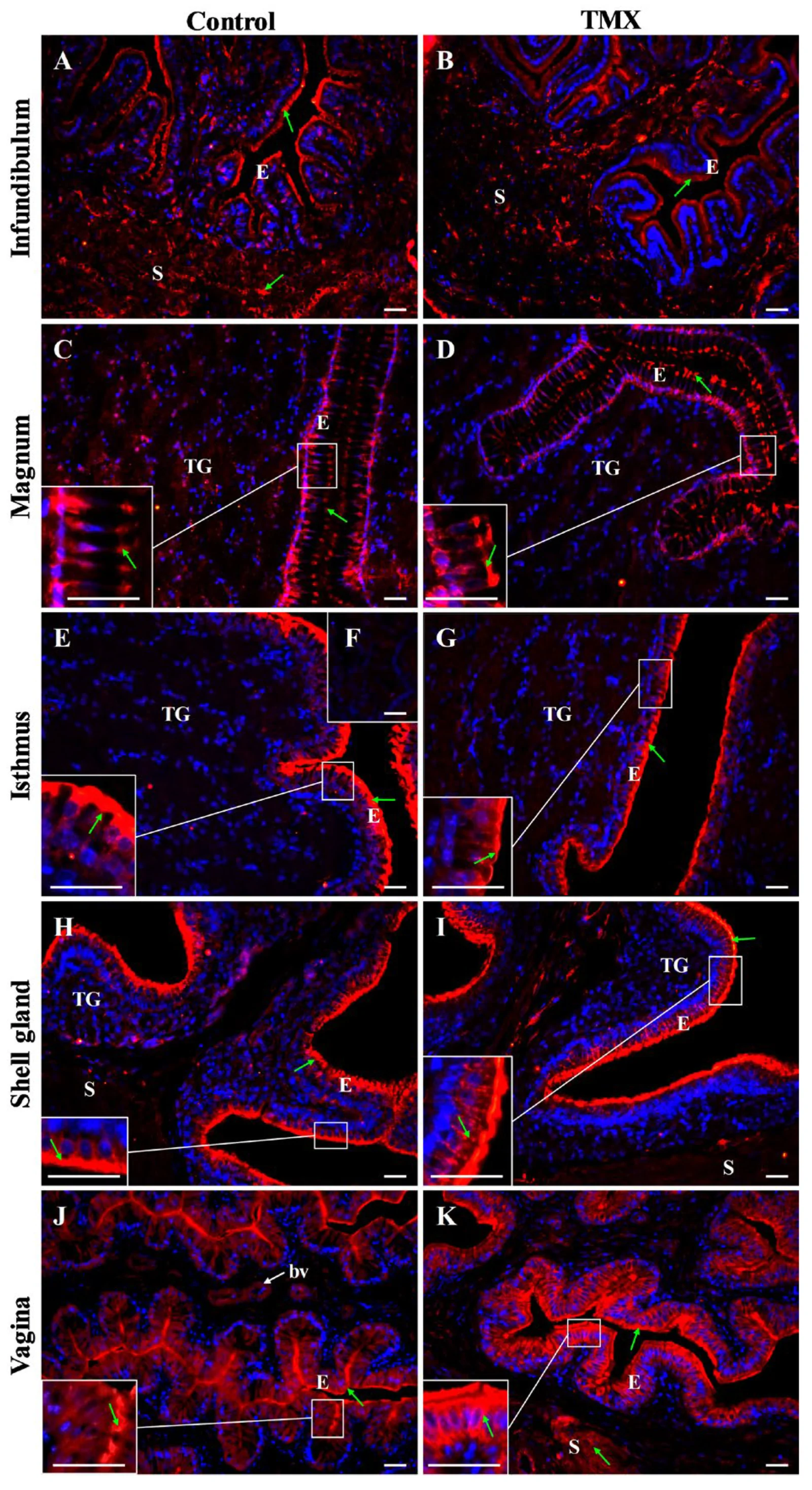
Immunofluorescent localization of E-cadherin protein in oviductal segments of control hens (**A**,**C**,**E**,**H**,**J**) and tamoxifen (TMX)-treated hens (**B**,**D**,**G**,**I**,**K**). Positive immunoreactive signals (red) are indicated by green arrows. DAPI staining (blue) indicates cell nuclei. Frames in (**C**–**E**,**G**–**K**) indicate the location of a higher magnification view. (**F**) Negative control (inset in (**E**)). In hens treated with TMX, E-cadherin signal intensity was diminished and redistributed from the cell membrane to the cytoplasm, with irregular staining compared to controls. Abbreviations: bv, blood vessel. Other abbreviations as in Figure 6. Scale bars = 20 μm.

**Figure 11:**
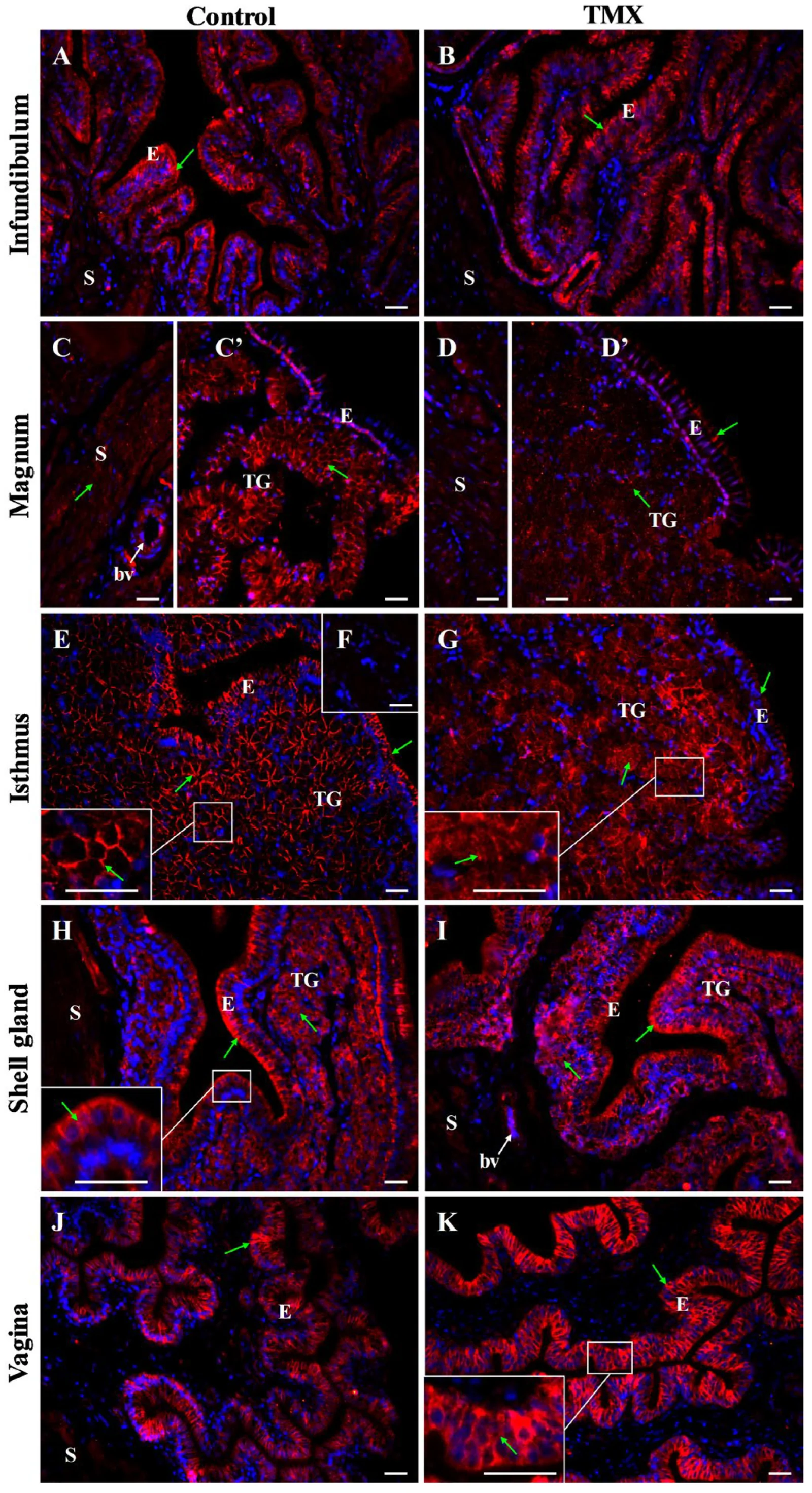
Immunofluorescent localization of β-catenin protein in oviductal segments of control hens (**A**,**C**,**C′**,**E**,**H**,**J**) and tamoxifen (TMX)-treated hens (**B**,**D**,**D′**,**G**,**I**,**K**). Positive (red) signals are indicated by green arrows. DAPI staining (blue) shows cell nuclei. The frames in (**E**,**G**,**H**,**K**) indicate the location of a higher magnification view (insets). (**F**) Negative control (inset in (**E**)). In TMX-treated hens, β-catenin signals were redistributed from the apical site to the cytoplasm, compared to controls. Abbreviations: bv, blood vessel. Other abbreviations as in Figure 6. Scale bars = 20 μm.

In line with the Western blot results, immunofluorescent analysis revealed the presence of E-cadherin and β-catenin proteins in all oviductal segments (Figure 10 and Figure 11). In control hens, strong immunopositive signals of E-cadherin were observed in the apical side of the luminal epithelium of the infundibulum, isthmus, shell gland, and vagina. Moderate immunoreactivity was detected in the luminal epithelium of the magnum and in stromal cells of the infundibulum, whereas very weak signals were present in tubular glands and stromal smooth muscles (Figure 10). Following TMX treatment, E-cadherin immunoreactivity became more concentrated in the apical region of luminal epithelial cells, with the staining pattern appearing more diffuse and irregular compared to the control group (Figure 10).

β-catenin immunoreactivity was detected in all oviductal segments (Figure 11). In control hens, strong immunopositive signals were observed in the cell membranes of luminal epithelial cells in the infundibulum, isthmus, shell gland, and vagina. Distinct linear signals clearly delineating cell borders were detected in the tubular glands of the magnum, isthmus, and shell gland, as well as in the blood vessels of the magnum. Moderate β-catenin immunoreactivity was observed in the luminal epithelium of the magnum. Weak or very weak signals were present in the smooth muscles of the infundibulum, magnum, shell gland, and vagina. Following TMX treatment, β-catenin immunoreactivity was differentially altered depending on the oviductal segment. Increased immunopositive signals were observed in the luminal epithelium of the infundibulum, shell gland, and vagina, whereas reduced immunoreactivity was detected in the luminal epithelium of the magnum and isthmus, compared to control hens. In the magnum, isthmus, and shell gland, the tubular glands showed marked disorganization following TMX treatment, with immunopositive signals appearing irregular and fragmented, in contrast to the distinct, linear patterns observed in control hens.

Negative control sections that were incubated without the primary antibody showed no immunopositive signals for E-cadherin or β-catenin (see inserts in Figure 10F and Figure 11F).

## 3. Discussion

The present study has demonstrated: (1) the transcription of genes encoding tight junction proteins (ZO1) and adherens junction proteins (*CDH1* and *CTNNB1*), as well as (2) the protein abundance and immunofluorescent localization of occludin, claudin 1, ZO1, E-cadherin, and β-catenin in oviductal segments of laying and non-laying (TMX-treated) hens. A pause in laying following TMX treatment was accompanied by significant changes in the mRNA and/or protein abundances, and in cellular distribution, of analyzed cell–cell junction proteins. Our findings suggest that tight and adherens junction proteins contribute to orchestrating the oviductal function and epithelial integrity, and their gene transcription and protein abundances are monitored by estrogen-dependent signaling pathways, in accord with previous results on a key gap junction protein, Cx43, in the hen oviduct following TMX treatment [19]. Furthermore, intercellular junction proteins have been identified in the oviduct [41,42] and ovary [43] of hens during a pause in laying induced by fasting. These proteins have also been shown in the turkey testes and epididymis [44] and the gander testes [45]. Our findings also largely align with observations in mammalian species and humans, where occludin, claudins, ZO1, E-cadherin, and β-catenin play a role in epithelial organization, barrier integrity, and function of the reproductive system [46,47,48,49,50]. Ultimately, our data bridge a critical gap by providing the first comprehensive map of tight and adherens junctions across the entire oviduct of hens.

First, in this study, histological and Feulgen staining confirmed that TMX treatment through several continuous days induces partial regression of the hen oviduct, manifested by epithelium thinning, glandular involution, and the appearance of condensed and fragmented nuclei, particularly in the magnum and isthmus. These features are also characteristic of degenerative and apoptotic processes accompanying tissue regression, as have been previously demonstrated in the hen oviduct following TMX treatment [13]. Since epithelial integrity is highly dependent on properly assembled intercellular junctions, the observed structural degeneration may be directly associated with destabilization of tight and adherens junction organization. Therefore, subsequently in the study, proteins comprising these cell–cell junctions were examined in the hen oviduct.

We found the oviductal segment-dependent expression of tight and adherens junction proteins at both the mRNA transcript and protein levels following TMX treatment. Among the analyzed mRNA transcripts, *ZO1* and *CTNNB1* mRNA levels exhibited segment-specific downregulation in response to TMX, whereas *CDH1* expression remained unaffected by TMX treatment. Similarly, the protein abundance of occludin, claudin 1, ZO1, E-cadherin, and β-catenin differed between segments in response to TMX treatment. In most cases, there was a decrease in protein abundance in the infundibulum, shell gland, and/or vagina. These segment-specific reactions may be attributed to distinct tissue compositions, physiological functions, and specific roles of individual junctional proteins in maintaining the organization of the epithelium and the integrity of the tissue within the oviduct, as well as variations in local estrogen receptor abundances [6,8]. The observed discrepancy in mRNA transcript and protein abundances for cell–cell junction proteins (ZO1, E-cadherin, β-catenin) in oviductal parts may be related to their posttranscriptional and/or posttranslational modifications, differences in translational efficiency, altered protein stability, or changes in protein turnover during tissue regression [51,52,53,54,55]. To establish the precise mechanisms of posttranscriptional and posttranslational modifications, as well as the importance of these modifications in the hen oviduct regression, further experiments are necessary. In particular, the use of techniques, such as nanopore direct RNA sequencing (DRS), which can capture multiple RNA modifications, full-length transcript architecture, alternative splicing patterns, and poly(A) tail features within individual molecules simultaneously, appear to be valuable in mechanistically interpreting the obtained results [56]. Furthermore, the use of the techniques for functional knockout of genes and their products may be important for elucidating the role of each specific gene in the avian oviduct. Interestingly, two distinct β-catenin immunoreactive bands were detected by Western blot analysis in both the control group and the TMX-treated group. As a central component of the Wnt/β-catenin signaling pathway, β-catenin is subject to continuous and extensive posttranslational modifications. Specifically, cytoplasmic β-catenin is phosphorylated by the destruction complex, including GSK-3β, which targets the protein for proteasomal degradation and slightly alters its electrophoretic mobility [57,58]. To establish if the additional band of β-catenin is unspecific or modified/phosphorylated remains to be elucidated. Under physiological conditions, a significant portion of β-catenin is localized at the cell membrane, where it contributes to the maintenance of tissue architecture through its association with adherens junctions. Released from adherens junction complexes, β-catenin can accumulate in the cytoplasm, where it undergoes continuous phosphorylation and degradation. Thus, altered intracellular distribution of β-catenin observed during TMX-induced oviduct regression in hens may result in a disruption of intercellular junctions and, eventually, organization of the luminal epithelium. Taken together, our findings provide evidence that estrogen receptor blockade by TMX affects intercellular junction proteins throughout the hen oviduct, resulting in remodeling of epithelial structure and function.

The pronounced alterations in examined protein abundances observed in the shell gland and vagina may reflect the exceptional specialization of these oviductal parts. The shell gland is characterized by intense ion transport and secretory activity associated with eggshell biomineralization, and the vagina is considered essential for epithelial barrier regulation to protect against ascending pathogens [40,59]. Therefore, blockage of the estrogen signaling may affect junctional organization, particularly in these highly specialized epithelial tissues.

More specifically, in this study, we observed a decrease in *ZO1* mRNA expression in the isthmus and shell gland of TMX-treated hens, concomitant with an increase in ZO1 protein abundance in the shell gland and vagina of TMX-treated hens. Slightly different observations were reported previously [41]. Namely, *ZO1* mRNA transcript and ZO1 protein abundances in the shell gland increased during a pause in laying induced by fasting. The discrepancy may be related to the factor inducing a pause in laying and physiological state of hens. However, the changes in ZO1 gene and/or protein expression, particularly in the shell gland, may indicate involvement of ZO1 protein in tight junction remodeling during TMX-induced oviduct regression. As ZO1 interacts with transmembrane junction proteins and the actin cytoskeleton, changes in its abundance may also affect overall junctional organization [60].

The protein abundance of another tight junction protein, claudin 1, increased in the infundibulum but decreased in the shell gland and vagina of TMX-treated hens compared to control hens. Similarly, Ariyadi et al. [40] demonstrated a downregulation of *CLDN 1*, *3*, and *5* mRNA in the isthmus, shell gland, and vagina of molting hens with a disrupted mucosal barrier. This effect was reversed after estrogen treatment.

Regarding adherens junction proteins, E-cadherin and β-catenin, treatment with TMX decreased E-cadherin protein abundance in the infundibulum and shell gland, as well as reduced β-catenin gene and protein expression in the infundibulum, whereas it augmented the abundance of this protein in the shell gland and vagina. β-catenin may act as both a structural component of adherens junctions and a regulator of intracellular Wnt signaling pathways that control epithelial function [24,61]. As cytoplasmic accumulation of β-catenin can precede nuclear translocation and activation of Wnt-related transcriptional pathways, the altered expression of β-catenin following TMX treatment may also reflect changes in intracellular signaling that resulted in epithelial remodeling and tissue regression.

The most important finding of the present study is the observation of altered immunoreactivity for proteins forming tight and adherens junctions and modifications in the signal patterns throughout the entire, partially regressed oviduct of TMX-treated, non-laying hens compared to laying birds. Immunofluorescent analysis revealed the localization of claudin 1, occludin, ZO1, E-cadherin, and β-catenin primarily in the luminal epithelium and, to a lesser extent, in the tubular glands of the hen oviduct. In control hens, immunopositive signals frequently appeared as continuous linear or punctate structures that clearly defined the borders between adjacent epithelial cells. This is consistent with the presence of properly organized tight and adherens junction complexes [62,63]. The strong immunoreactivity for claudin 1, ZO1, E-cadherin, and β-catenin was observed in the shell gland and vagina. In these segments, the signals often delineated the borders of epithelial cells, suggesting that junctional organization plays an important role in maintaining the integrity of the epithelium and regulating transport in the distal oviductal segments that are involved in eggshell formation and protection against pathogens [64]. Distinct immunoreactivity was also observed in the tubular glands of the magnum, isthmus, and shell gland, where signals frequently formed organized linear patterns. As the magnum synthesizes and secretes most of the egg albumen, the isthmus forms the inner and outer eggshell membranes, and the shell gland adds electrolytes to the albumen and forms the calcified eggshell [1,2], the observed localization pattern suggests that properly assembled junctional proteins contribute to maintenance of epithelial polarity and coordinated secretory activity. The strong ZO1 and E-cadherin immunoreactivity observed in the smooth muscle of the infundibulum of control hens was particularly notable. This suggests that cell–cell junction proteins in the oviduct of hens may play a role not only in forming an epithelial barrier but also in coordinating the muscle activity required for capturing the ovum, egg transport through the oviduct, and oviposition. This is similar to the previously proposed role for Cx43 in the oviduct of hens [19]. Of all the proteins that were analyzed, β-catenin displayed the broadest cellular distribution, being present in epithelial cells, tubular glands, smooth muscle layers, and blood vessels. Our results are consistent with those of Bae et al. [65], who demonstrated strong β-catenin expression in the luminal epithelium of the chicken oviduct and in the tubular glands of the oviductal isthmus and shell gland. This widespread localization of β-catenin suggests that its functions may extend beyond the role of cell adhesion, which is consistent with its established involvement in signaling pathways that are responsible for maintaining epithelial tissue [66,67]. As tight and adherens junctions are crucial for epithelial cohesion, polarity, vectorial secretion, and cellular homeostasis, their disruption could lead to increased paracellular permeability, epithelial instability, and activation of apoptotic pathways. Therefore, the redistribution of junctional proteins towards the cytoplasm and apical compartments likely reflects impaired epithelial polarization and disturbed secretory coordination within the regressing oviduct after TMX treatment. In particular, the altered organization of the E-cadherin/β-catenin complex may affect structural adhesion and the intracellular signaling involved in cell survival and differentiation [68,69], potentially contributing to the glandular involution.

Following TMX treatment, the spatial organization of junctional proteins was disrupted, characterized by weaker and more fragmented immunopositive signals, loss of clearly defined epithelial borders, and redistribution toward the cytoplasmic or apical regions of luminal epithelial cells. These alterations were accompanied by diffuse staining and structural disorganization of tubular glands, which are part of the magnum, isthmus, and shell gland. The changes were most evident with regard to occludin, ZO1, E-cadherin, and β-catenin proteins and are generally considered to be indicators of junctional destabilization and impaired epithelial organization [70,71,72,73]. Similar changes have previously been associated with epithelial destabilization, increased paracellular permeability, and reduced cell–cell adhesion in barrier-forming epithelial tissues [28,74]. Furthermore, the relocation of ZO1 and E-cadherin proteins from the plasma membrane may suggest disrupted interactions with the actin cytoskeleton, which are crucial for stabilizing and maintaining epithelial architecture [21]. Our results indicate that TMX affects both the total abundance of proteins, as assessed by Western blot, and their spatial organization, leading to remodeling of tight and adherens junctions. In turn, altered/disrupted cell–cell junctions may weaken the mechanical stability of the epithelium and increase its susceptibility to regression [75], as well as affect glandular organization and secretory function. Overall, our results support the hypothesis that the maintenance of junctional integrity critically depends on estrogen action and represents a key component of normal oviduct structure and function in hens.

## 4. Materials and Methods

### 4.1. Animals, Experimental Design, and Tissue Sampling

Hy-Line Brown laying hens were obtained from a commercial breeder at 32 weeks of age. They were housed individually under controlled conditions (14L:10D photoperiod) and had unrestricted access to commercial feed and water. After a two-week acclimatization period, hens were randomly allocated into two groups (n = 6 birds per group): a control group (C) and a tamoxifen-treated group (TMX). The experimental group received daily subcutaneous injections of TMX (6 mg/kg body weight, dissolved in 0.3 mL of ethanol; Sigma-Aldrich, St. Louis, MO, USA), while the control hens received the corresponding volume of the vehicle solution as previously described by Socha and Hrabia [13], Hrabia et al. [19], and Frydrych et al. [15]. The hens were treated daily (in the morning) for 7 consecutive days of the experiment until a pause in egg laying occurred in all TMX-treated birds. On day 8 of the experiment, the hens were humanely euthanized. The TMX-treated birds were sacrificed 2–3 days after the last oviposition, while the control hens were euthanized approximately 2–2.5 h post-oviposition (when plasma steroid concentrations are relatively low) to ensure a comparable physiological state. The oviducts were immediately harvested and placed on ice. All oviductal parts were isolated: the infundibulum, magnum, isthmus, shell gland, and vagina. Tissue samples were collected from the middle portion of each segment per oviduct/hen. For molecular analyses, samples were snap-frozen in liquid nitrogen and stored at −80 °C or preserved in RNAlater for subsequent RNA isolation. Additional tissue fragments were fixed in freshly prepared 10% buffered formalin (0.1 M phosphate buffer, pH 7.6), processed routinely, and embedded in paraffin wax for further histological and immunofluorescent analyses.

### 4.2. Total RNA Isolation and Reverse Transcription

Total RNA extraction and reverse transcription (RT) were performed using methods previously described by Hrabia et al. [19] and Frydrych et al. [15,43]. In brief, total RNA was extracted from oviductal tissue samples originating from five different randomly chosen hens/oviducts of each group (control and TMX-treated) using TRI reagent (Sigma-Aldrich). Then, 2 μg of total RNA from each sample was reverse transcribed using a High-Capacity cDNA Reverse Transcription Kit (Thermo Fisher Scientific, Waltham, MA, USA) and random primers. The reaction was carried out in a final volume of 20 μL, according to the manufacturer’s protocol: 10 min at 25 °C, 120 min at 37 °C, and 5 min at 85 °C. A pooled RNA sample derived from all oviductal segments and experimental groups was processed without reverse transcriptase to verify the absence of genomic DNA contamination.

### 4.3. Quantitative Real-Time PCR (qRT-PCR)

Following a 10-fold dilution, the obtained cDNA was used for quantitative real-time PCR (qRT-PCR) with chicken-specific TaqMan Gene Expression Assays for *ZO1*, *CDH1*, and *CTNNB1* (Table 1) on a QuantStudio 3 Real-Time PCR System (Applied Biosystems, Waltham, MA, USA, Thermo Fisher Scientific), as previously described [19,41,43]. The cycling parameters were as follows: 2 min at 50 °C, followed by 10 min at 95 °C, and 40 cycles of 15 s at 95 °C and 60 s at 60 °C. Duplex qRT-PCR was performed for each target gene together with the reference gene, *18S rRNA*, in a 10 μL reaction volume containing: 5 μL of TaqMan Gene Expression Master Mix (Applied Biosystems by Thermo Fisher), 0.5 μL of TaqMan Gene Expression Assay (Applied Biosystems by Thermo Fisher) containing a specific minor groove binding (MGB) probe and primers, 0.5 μL of Eukaryotic *18S rRNA* Endogenous Control (established as an appropriate control for oviductal tissues and TMX treatment; VIC/TAMRA -TaqMan probe), 3 μL of water, and 1 μL of cDNA. Both target and control gene amplification efficiency was in the range 95–103%. Each sample was analyzed in duplicate, and a no-template control was included in each run. The relative quantity (RQ) of transcripts was calculated after normalization to the *18S rRNA* transcript, and expression in the tissue of the control group used as a calibrator was applied using the 2^−ΔΔCt^ method [76]. The consistency of technical replicates was assessed based on the absolute difference between Ct values. Replicates exceeding the predefined acceptance threshold of 0.5 Ct were excluded from the calculations.

### 4.4. Protein Extraction and Western Blot Analysis

Analyses were performed to determine the protein abundance of occludin, claudin 1, ZO1, E-cadherin, and β-catenin in oviductal tissue samples. Briefly, tissue samples collected from six hens/oviducts from each group (control and TMX-treated) were crushed in liquid nitrogen and lysed in an ice-cold lysis buffer (BioVision, Milpitas, CA, USA). The homogenates were then sonicated and centrifuged at 10,000× *g* for 20 min at 4 °C. The protein concentration in the resulting supernatant was determined using the Bradford assay with the Pierce Detergent Compatible Bradford Assay Reagent (Thermo Scientific, Rockford, IL, USA), with bovine serum albumin used as the standard. According to previously described procedures [19,41,42,43], equal amounts of protein (60 µg) were denatured in Laemmli buffer (Bio-Rad, Hercules, CA, USA) at 99 °C for 7 min and separated by SDS-PAGE under reducing conditions. The gel concentration was selected depending on the analyzed protein: 12% for occludin and claudin 1, 10% for E-cadherin, and 8% for ZO1 and β-catenin. Electrophoresis was performed using a Mini-Protean Tetra Cell system (Bio-Rad). The proteins were then transferred onto PVDF membranes using a semi-dry transfer system in FLASHBlot buffer (Advansta, San Jose, CA, USA) at 25 V for 7 min. The membranes were then blocked for 1 h in Tris-buffered saline containing 0.1% Tween 20 (TBST, pH 7.6) supplemented with 5% non-fat dry milk. After washing, the membranes were incubated overnight at 4 °C with primary rabbit polyclonal antibodies against occludin, claudin 1, ZO1, E-cadherin, and β-catenin (Table 2). The specificity of these antibodies had previously been confirmed in avian tissues [41,42,43,44,45]. Following incubation, the membranes were washed and incubated with horseradish peroxidase-conjugated goat anti-rabbit secondary antibodies for 1 h. Immunoreactive bands were visualized using an enhanced chemiluminescence detection system (WesternBright™ ECL, Advansta, San Jose, CA, USA). A ChemiDoc-It 410 imaging system (Ultra-Violet Products Ltd., Cambridge, UK) with VisionWorks Life Science software (version 6.6a) was used to acquire the signal. Subsequently, the membranes were stripped and reprobed with anti-β-actin or anti-β-tubulin antibodies to serve as loading controls. All analyses were performed in quadruplicate. Protein bands were quantified densitometrically using ImageJ software (version 1.8.0, National Institutes of Health, Bethesda, MD, USA), and the results were normalized to the corresponding loading control.

### 4.5. Histological and Immunohistochemical Analysis

#### 4.5.1. Hematoxylin and Eosin Staining

Hematoxylin and eosin (H&E) staining was performed to evaluate the structural organization of the oviductal wall. Paraffin-embedded tissue fragments were cut to a thickness of 3 µm and mounted on slides coated with 3′3′-aminopropyltriethoxysilane. Following deparaffinization and rehydration, the sections were stained with Harris hematoxylin (Epredia, Portsmouth, NH, USA) for 5 min, differentiated in 1 M HCl for 1 s, and counterstained with eosin Y (Epredia) for 30 s. The slides were then dehydrated in ethanol, cleared in xylene, and mounted with DPX mounting medium (Sigma-Aldrich) under a coverslip. Microscopic analysis was performed using an Axio Scope.A1 light microscope (Carl Zeiss, Oberkochen, Germany), and images were acquired using a ZEN 2.3 Pro software-enabled Axiocam 503 color camera (Carl Zeiss). The thickness (µm) of the luminal epithelium was measured independently by two observers in two cross-sections of each oviductal part, and counts were averaged per bird. The bird-level means (n = 6 per group) were used for statistical analysis.

#### 4.5.2. Feulgen Staining

Feulgen staining was performed according to protocols previously described [12,41]. This method selectively stains cell nuclei, enabling the visualization of DNA distribution within tissues. In brief, following deparaffinization and rehydration, the sections (6 µm thick) were hydrolyzed in 1 M HCl at 60 °C for 12 min, after which they were incubated with Schiff’s reagent (Sigma-Aldrich) in the dark for 30 min. The reaction was terminated by rinsing in 10% Na_2_SO_4_ for 15 s, followed by washing in running water for 3 min. The sections were then dehydrated and mounted with DPX. Slides were examined using the same microscopy setup as for H&E staining.

#### 4.5.3. Immunofluorescence

Immunofluorescent analysis was performed to determine the localization of occludin, claudin 1, ZO1, E-cadherin, and β-catenin in the wall of the oviducts collected from three randomly chosen hens from each group, following protocols previously established by Hrabia et al. [19], Frydrych et al. [15,41,43], and Frydrych and Hrabia [42]. In brief, paraffin sections (6 µm thick; two sections/oviductal segment/hen/group) were deparaffinized in xylene, rehydrated through a series of graded ethanol solutions, and rinsed in distilled water. Antigen retrieval was performed by heating tissue sections in a microwave in citrate buffer (pH 6.0) for 3 min. Non-specific binding was blocked using 5% normal goat serum in TBST for 30 min at room temperature. The sections were then incubated overnight at 4 °C with primary rabbit polyclonal antibodies (Table 2). After washing in TBS, the sections were incubated for 90 min with DyLight 594-conjugated anti-rabbit IgG. The nuclei were then counterstained with DAPI in VECTASHIELD^®^ Vibrance™ Antifade Mounting Medium (Vector Laboratories, Burlingame, CA, USA), and the slides were coverslipped. Negative controls were prepared by replacing the primary antibody with normal rabbit serum, as well as with TBST. Oviductal sections (three–four sections/oviductal segment/three hens/group) were analyzed by two independent observers blinded to the treatment groups using an Axio Scope.A1 fluorescence microscope (Carl Zeiss), and images were captured using a ZEN 2.3 Pro software-controlled Axiocam 503 color camera (Carl Zeiss). Immunoreactivity was assessed semi-quantitatively as strong, moderate, weak, or very weak. Representative images show merged DAPI (blue) nuclear staining and DyLight 594 (red) fluorescence corresponding to the localization of specific proteins.

### 4.6. Statistical Analysis

The variables were examined for normality and homogeneity of variance using the Shapiro–Wilk and Brown–Forsythe tests, respectively. Since the data on the thickness of the luminal epithelium in the oviduct of the control group (Figure 3) passed the tests for normality (*p* < 0.05) and homogeneity of variance (*p* < 0.05), they were examined by one-way ANOVA (effect of oviductal section), followed by the Tukey test, which incorporates an FWER correction for multiple comparisons. For comparison of the means of the TMX-treated vs. control group (effect of treatment), Student’s *t*-test was used when data pass normality and homogeneity of variance (Figure 3), while the nonparametric Mann–Whitney U test was used when data did not pass normality and/or homogeneity of variance (Figure 4, Figure 5 and Figure 6). Differences in values were considered significant at the 95% confidence level (*p* < 0.05). Calculations were performed using SigmaPlot_V13 (Systat Software, Inc., San Jose, CA, USA). The results are presented as the mean ± standard error of the mean (SEM).

## 5. Conclusions

In summary, changes in the tight (claudin 1, occludin, ZO1) and adherens (E-cadherin, β-catenin) junction protein gene and/or protein expression (Table 3) and the tissue distribution of immunopositive signals were shown in oviductal parts of TMX-treated (non-laying) hens compared to laying hens. Thus, the results demonstrated, in large parts for the first time, point out the potential participation of these proteins in the orchestration of intercellular communication, cell signaling, paracellular permeability, and mucosal barrier functionality, which affect the hen oviduct function. Furthermore, obtained results provide novel insights into the importance of estrogen in the regulation of these protein expressions in the avian oviduct. Further studies are warranted to establish the impact of changes in junctional protein organization on epithelial function and their regulation by steroid hormones in the hen oviduct. That may be of considerable importance for poultry production.

## Figures and Tables

**Table 1:** GenBank accession numbers, assay ID, and amplicon length obtained using a quantitative real-time polymerase chain reaction (qRT-PCR) assay for chicken tight and adherens junction-related genes.

Gene	GeneBank Identification	Assay ID/Cat. No.	Amplicon Size [bp]
*ZO1*	XM_015278975.2	Gg07169479_m1	88
*CDH1*	NM_001039258.2	Gg03357892_g1	83
*CTNNB1*	NM_205081.2	Gg03348506_g1	84
*18S rRNA*	X03205.1	Cat. no. 4310893E	187

Abbreviations: *ZO1*, zonula occludens protein 1 (also known as tight junction protein 1 [TJP1]); *CDH1*, E-cadherin; *CTNNB1*, β-catenin; rRNA, ribosomal RNA.

**Table 2:** List of primary and secondary antibodies used for Western blot (WB) and immunofluorescence (IF).

Antibody	Serum	Host Species	Vendor	Cat. No	WB Dilution	IF Dilution
Claudin 1	NGS	Rabbit	Invitrogen, Carsband, CA, USA	51-9000	1:500	1:75
Occludin	NGS	Rabbit	Invitrogen, Carsband, CA, USA	71-1500	1:250	1:75
Zonula occludens 1	NGS	Rabbit	Invitrogen, Carsband, CA, USA	61–7300	1:500	1:50
E-cadherin	NGS	Rabbit	Invitrogen, Carsband, CA, USA	PA5-19479	1:1000	1:100
Beta-catenin	NGS	Rabbit	Invitrogen, Carsband, CA, USA	71-2700	1:1000	1:75
Goat anti-rabbit HRP	-	Goat	Advansta, San Jose, CA, USA	R-05072-500	1:5000	-
Anti-beta-actin HRP	-	Mouse	Sigma-Aldrich, St. Louis, MO, USA	A2228	1:500	-
Anti-beta-tubulin	-	Mouse	Invitrogen, Carsband, CA, USA	MA5-16308	1:2500	-
DyLight 594-anti-rabbit	-	Goat	Vector Laboratories, Burlingame, CA, USA	DI-1594-1.5	-	1:150

Abbreviations: HRP, horseradish peroxidase; NGS, normal goat serum.

**Table 3:** Summary of TMX treatment effects on gene and protein expression of tight and adherens junction proteins in the hen oviduct.

Oviductal Segment	Gene/Protein							
	Claudin 1	Occludin	*ZO1*/ZO1		*CDH1*/E-Cadherin		*CTNNB1*/β-Catenin	
	Protein	Protein	mRNA	Protein	mRNA	Protein	mRNA	Protein
Infundibulum	**↑**	**↓↓↓**	**–**	**↓↓↓**	**–**	**↓↓**	**↓**	**↓↓↓**
Magnum	na	na	**–**	na	**–**	na	**↑**	na
Isthmus	na	na	**↓**	na	**–**	na	**–**	na
Shell gland	**↓↓↓**	**–**	**↓↓**	**↑↑↑**	**–**	**↓↓↓**	**–**	**↑↑↑**
Vagina	**↓↓↓**	**↓↓↓**	**–**	**↓↓↓**	**–**	**–**	**–**	**↑↑↑**

Abbreviations as in Table 1. Explanations: Red arrows indicate decreased mRNA transcript level or protein abundance, whereas green arrows indicate increased mRNA transcript level or protein abundance. One, two, and three arrows indicate statistical significance at *p* < 0.05, *p* < 0.01, and *p* < 0.001, respectively; – indicates no significant change; na, not analyzed.

## Data Availability

The raw data used for the preparation of the presented results are available on request from the corresponding author.
